# Supplementary material for: Monitoring risk assessment on an acute psychiatric ward: Effects on aggression, seclusion and nurse behaviour
Source: PLoS One. 2020 Oct 2;15(10):e0240163. doi: 10.1371/journal.pone.0240163 (PMC7531854; doi:10.1371/journal.pone.0240163)
Supplement: S8 File — (DOCX) [file pone.0240163.s008.docx]

Read me first.

This is an appendix to the data files with explanation and translations for correct interpretation and reading.

- Data file “data patiens”

| *Dutch* | Subjectnummer | Geboortedatum | Geslacht | Etniciteit | Opnamedatum | Juridische maatregel | Ontslagdatum | Totale opnameduur | Diagnose |
| --- | --- | --- | --- | --- | --- | --- | --- | --- | --- |
| *English* | Subject number | Date of birth | Sex | Ethnicity | Date of admission | Legal measures | Date of dismissal | Total duration of admission | Diagnosis |
|  |  |  | 1=male  2=female | 1=Dutch  2=Western  3=Non-Western |  | 1=Voluntary  2= Court authorization  3= Custody measure |  |  | *1=Psychosis 2=Personality disorder*  *3= substance abuse*  *4=other primary diagnosis* |

- Data file “data nurses”

| *Dutch* | Meetmoment | Subjectnr | Geboortedatum | Leeftijd | Geslacht | Opleiding | Werkervaring vpk | Werkervaring hic |
| --- | --- | --- | --- | --- | --- | --- | --- | --- |
| *English* | Moment of measurement | Subject number | Date of birth | Age | Sex | Education | Work experience as nurse (years) | Work experience as nurse at high intensive care (psychiatry) (years) |
|  | 0=baseline  1=post implementation |  |  |  | 1=male  2=female | 4=mbo*  5=havo/vwo*  6=hbo*  7=wo*  * Dutch education system |  |  |

- Data file “coercive interventions”

| *Dutch* | Meetfase | Subjectnr | Interventie | Sepduur in min | Akkoord |
| --- | --- | --- | --- | --- | --- |
| *English* | Moment of measurement | Subject number | Intervention | Duration seclusion in minutes | Agreement |
|  | 0=baseline  1=post implementation |  | 0=Forced medication  1=Seclusion  2=Seclusion EBK  3=Intensive care unit, one-on-one treatment  4= fixation |  | 0=coercion  1=agreement with intervention  2=on request of patient |

- Data file “SDAS scores total”

| *Dutch* | Meetmoment | Subjectnummer | Weeknummer |
| --- | --- | --- | --- |
| *English* | Moment of measurement | Subject number | Week number |
|  | 0=baseline  1=post implementation |  |  |

- Data file “Extra analysis coercive interventions and stress”

| *Dutch* | Meetmoment | Datum | Dag | Sepduur  (EBK+sep) in min | Sep aanvang ‘snachts (>22.30-07.00); aantal | Noodmedicatie | Med aanvang ’s nachts  (>22.30-07.00); aantal | Stressniveau | Weekend |
| --- | --- | --- | --- | --- | --- | --- | --- | --- | --- |
| *English* | Moment of measurement | Date | Day | Duration of seclusion in minutes | Seclusion at night ; number | Involuntary, emergency medication; number | Involuntary, emergency medication at night; number | Stress level | Weekend |
|  | 0=baseline  1=post implementation | Day-month-year | Maandag=Monday  Dinsdag=Tuesday  Woensdag=Wednesday  Donderdag=Thursday  Vrijdag=Friday  Zaterdag=Saturday  Zondag=Sunday |  |  |  |  |  | 0=no  1=yes |

|  |  |  |  |  |  |  |  |  |
| --- | --- | --- | --- | --- | --- | --- | --- | --- |
