# Supplementary material for: Monitoring risk assessment on an acute psychiatric ward: Effects on aggression, seclusion and nurse behaviour
Source: PLoS One. 2020 Oct 2;15(10):e0240163. doi: 10.1371/journal.pone.0240163 (PMC7531854; doi:10.1371/journal.pone.0240163)
Supplement: Attachment — Submitted filename: Rebuttal letter_200512.docx [file pone.0240163.s009.docx]

E JR Florisse

Mondriaan, Mental Health Care

John F Kennedylaan 301

6419 XZ Heerlen

The Netherlands

[e.florisse@mondriaan.eu](mailto:e.florisse@mondriaan.eu)

Editor-in-Chief

*PLOS ONE*

April 9, 2020

Dear Editor of *PLOS ONE*,

We thank the reviewers for their positive feedback and the comments on the manuscript. We have edited the manuscript to address their concerns.

Reviewer#1 gave minor but valuable suggestions on grammar. All suggestions from this reviewer were implemented and corrected in the manuscript:

- *Remove 'behavioural' after changes on line 62.*
- *Remove 'the' before patients (line 62) and before 'staff' (line 66). Same for line 74 (x2)*
- *Under 'Design' 'This study assessed' not 'assesses'.*
- *Remove 'long' after 26 weeks (line 97)*
- *Line 98 "Nursing staff were trained"*
- *Line 99, remove 'the' and start with "Monitoring....*
- *Under intervention, I was confused by the word 'taxation' should this be 'assessment'?*
- *Line 131, Suggest removing 'abuse' and replace with 'misuse'.*
- *Line 137 "The BVC was" not 'is'*
- *Line 145, suggest removing 'several times'.*
- *Line 151, suggest changing 'filled in' with 'completed'.*

Reviewer#2
We addressed the comments of reviewer#2 as follows:

- *I need for you to elaborate a bit more on how you have instructed the staff to use the crisis monitor. In line 137 you write about BVC. The way you describe is ok, but I think there is a lack in your description of how to use the BVC. As I understand from your description the BVC is only scored once a day. The official manual formulates that the BVC is scored once each shift (that would in your case be three times a day). You are missing a reference to the results of BVCs capabilities.*

*I also need for you to describe how you have instructed the staff to act after a score above the cut-off score on the BVC. If you use the BVC a standalone Risk assessment tool there is an agreement that a score on 3 or above triggers an intervention – I cannot read from your paper how you have instructed the staff about such thing. Because of these shortcomings I have some reservation about your conclusion. In risk-assessment research I think there is an agreement that the risk-assessment tool in itself is not enough it is the intervention therefore before I In your paper can see a description of how the staff is instructed to use their results og the score on the Crisis monitor I cannot accept the paper.*

We thank the reviewer for pointing to this need for clarification. We have made changes in the manuscript to be clearer about the instructions to the staff with regard to the use of the Crisis Monitor. The BVC was scored twice a day at the end of each shift (excluding night shift) and there were specific instructions when BVC was above cut-off. Also, ratings of the instruments were discussed on regular basis and if necessary, decisions were made to manage specific risks by introducing de-escalating interventions. Changes were made in line 111, 114, 115, 139 and 140 of the manuscript.

- *In your results from study 2 you have some significant results from baseline to post implementation “What are you doing” administration, “with whom” alone and “Where are you” Nursing post. I’m missing that you take into account that you ask the staff to both use a crisis monitor that increases the administration load and the you also disturb the staff with replying to the PsyMate – all in all that in itself gives staff an increased administrative workload and with an increased workload there is less time left for being with the patients – so maybe you have created for yourself an environment that lead to more aggression - just some thoughts for you to think about and to sharpen your discussion.*

Indeed, we believe the administration load of the staff is increased significantly due to implementation of the Crisis Monitor. That is the sole factor that is differed between baseline and post implementation. The burden of the PsyMate was equal in baseline and post implementation and could therefore not be responsible for the significant results. Also, by design the Crisis Monitor objectively took more time ((2shifts*5minutes*7days + 15minutes/week)*22patients*80%occupancy = averaging 1500 minutes/week) than the PsyMate (2shifts*3nurses*6beeps*90secs*7days = 378 minutes/week, but only one week out of 8 = averaging 48 minutes/week or 3%). Moreover, the PsyMate administrative time might even be underestimated in the recorded figures, since users are asked in the PsyMate to describe what they were doing before the beep signal. This excludes most PsyMate administrative time in the reports, and consequently the change in administrative time ratings are primarily due to the intervention, in casu, the Crisis Monitor. To be clearer on this point we made some changes in the manuscript in line 255, 256, 264, 265, 356-363.

Thank you very much for your consideration.

Yours sincerely,

E JR Florisse
